# Reduced Expression of Erythropoietin After Intravitreal Ranibizumab in Proliferative Diabetic Retinopathy Patients—Retrospective Interventional Study

**DOI:** 10.3389/fmed.2021.710079

**Published:** 2021-09-21

**Authors:** Li Chen, Jing Feng, Yanhong Shi, Fuxiao Luan, Fang Ma, Yingjie Wang, Weiqiang Yang, Yong Tao

**Affiliations:** Department of Ophthalmology, Beijing Chaoyang Hospital, Capital Medical University, Beijing, China

**Keywords:** erythropoietin, intraocular fluid, intravitreal ranibizumab, proliferative diabetic retinopathy (PDR), vascular endothelial growth factor (VEGF)

## Abstract

**Purpose:** To evaluate the expressions of erythropoietin (EPO) and vascular endothelial growth factor (VEGF) in the vitreous and fibrovascular membranes (FVMs) of proliferative diabetic retinopathy (PDR) after the intravitreal injection of ranibizumab (IVR) and further explore the relationship between EPO and VEGF.

**Method:** The concentrations of EPO and VEGF levels in the vitreous fluid were measured in 35 patients (24 PDR and 11 non-diabetic patients) using enzyme-linked immunosorbent assay. The patients were divided into three groups: PDR with IVR (IVR group) before par plana vitrectomy (*n* = 10), PDR without IVR (Non-IVR group) (*n* = 14) and a control group [macular holes (MHs) or epiretinal membranes (ERM), *n* = 11]. Fluorescence immunostaining was performed to examine the expressions of VEGF, EPO and CD 105 in the excised epiretinal membranes.

**Result:** The PDR eyes of Non-IVR group had the highest vitreous VEGF and EPO levels (836.30 ± 899.50 pg/ml, 99.29 ± 27.77 mIU/ml, respectively) compared to the control group (10.98 ± 0.98 pg/ml and 18.96 ± 13.30 mIU/ml/ml). Both the VEGF and EPO levels in the IVR group (13.22 ± 2.72 pg/ml and 68.57 ± 41.47 mIU/ml) were significantly lower than the Non-IVR group (*P* = 0.004 and *P* = 0.04, respectively). Furthermore, no significant difference was observed for VEGF levels between the control and IVR groups (10.9 ± 0.98 pg/ml and 13.22 ± 2.72 pg/ml, respectively, *P* = 0.9). Yet the EPO level in the IVR group was significantly higher than that in the Non-diabetic group (68.57 ± 41.47 pg/ml and 18.96 ± 13.30 pg/ml, respectively, *P* = 0.001). The expressions of EPO, VEGF, and CD105 were significantly reduced in fluorescence immunostaining of FVMs in the IVR group compared with the Non-IVR group. The receiver operating characteristic (ROC) curve of the EPO and VEGF levels were 0.951 and 0.938 in the PDR group.

**Conclusion:** Both of the VEGF and EPO level were significantly increased in PDR patients, which have equal diagnostic value in the prediction of PDR. IVR could reduce the EPO level, but not enough to the normal level.

## Introduction

Proliferative diabetic retinopathy (PDR) is advanced diabetic retinopathy (DR), characterised by the pathologic growth of new blood vessels, which is driven by the release of local angiogenic factors in ischemic and hypoxic retina (1). Vascular endothelial growth factor (VEGF) is a potent mediator that controls angiogenesis and vascular permeability in both pathological and physiological ocular conditions (2). The current evidence indicates that VEGF plays a central role in the development of DR (3–5). Although the inhibition of VEGF reduces retinal neovascularization, it does not completely inhibit ischemia-driven retinal neovascularization (6). Thus, the angiogenic process is likely to involve numerous growth factors and cytokines (7).

Erythropoietin (EPO) is a pleiotropic cytokine, with the function of a circulatory growth factor (8). Higher levels of EPO in the vitreous and serum samples of PDR patients than in a control group were recently demonstrated (9–12). Furthermore, the evidence shows that EPO is a potent retinal angiogenic factor independent of VEGF and is capable of stimulating ischemia-induced retinal angiogenesis in PDR (13). Evidences have proved that EPO has an angiogenic potential equal to VEGF. There is no information available regarding the influence of anti-VEGF for the EPO level.

The purpose of the present study was to evaluate the changes of VEGF and EPO vitreous concentrations after intravitreal ranibizumab injection and to detect the expressions of VEGF and EPO on epiretinal fibrovascular membranes (FVMs) obtained during vitrectomy in eyes with PDR.

## Patients and Methods

### Subjects and Enrolment Criteria

This was a retrospective, interventional study. The study was conducted in accordance with the Declaration of Helsinki, and we received approval from the Investigational Review Board of the Beijing Chaoyang Hospital (2018-4-3-3). Informed consent for all examinations and procedures was obtained from the patients, who provided their written informed consent to participate. This study enrolled patients with vitreous haemorrhage or tractional retinal detachment (TRD) as the PDR groups and non-diabetic patients with idiopathic macular hole (MH) or macular epiretinal membranes (ERM) as the control group. All the patients underwent pars plana vitrectomies (PPV) between January 2019 and June 2020. The inclusion criteria for the PDR group were type 2 diabetes, age > 18 years and PDR. The exclusion criteria were as follows: (1) any anti-VEGF therapy or pan-retinal photocoagulation within 6 months prior to the study; (2) any history of ocular diseases other than DR; (3) a history of ocular surgery on the study eye; and (4) a history of systemic thromboembolic events, including myocardial infarction and stroke. The exclusion criteria for the non-diabetic control group were uveitis, a previous intraocular surgery, diabetes mellitus, a malignant tumour and the use of immunosuppressive drugs.

Thirty-five patients (35 eyes) fulfilled the inclusion criteria and were divided into three groups: (1) 14 PDR patients underwent PPV without intravitreal ranibizumab (IVR) treatment (Non-IVR group); (2) 10 PDR patients underwent PPV with IVR treatment (0.5 mg/0.05 ml of intravitreal ranibizumab injected 7–10 days before surgery) (IVR group); and (3) 11 patients with MH or macular ERM as the control group.

### Physical and Ocular Examinations

Each patient's demographic, clinical, and ocular data were recorded. Each patient (diabetics and controls) underwent complete ophthalmological examinations, including visual acuity, slit lamp, tonometry, fluorescein retinal angiography, and optical coherence tomography. Diabetic retinopathy was evaluated using standardised fundus colour photographs and fluorescein angiograms. If a vitreous haemorrhage or lens opacity prevented an ophthalmoscopic examination of the ocular fundus, an ocular ultrasound was the auxiliary examination.

### Intravitreal Ranibizumab Injection

A 30-gauge needle was inserted through the corneal limbus to withdraw 0.05 mL of aqueous humour and to soften the globe. Subsequently, 0.5 mg (0.05 mL) of ranibizumab was injected into the vitreous fluid as preoperative adjunctive therapy 7 days before vitrectomy. Topical antibiotics were applied as postoperative medications.

### Surgical Procedures and Vitreous Sampling

All the surgeries were performed by the same surgeon at the Beijing Chaoyang Hospital. All the patients underwent a 23-gauge standardised technique pars plana vitrectomies. At the beginning of surgery, 0.5 mL of undiluted vitreous sample was aspirated through the vitreous cutter under simultaneous inflation of the vitreous cavity with air through the infusion cannula. For ethical and technical reasons, it was impossible to obtain paired samples of vitreous humour in the same eye (with and without IVR). Therefore, the vitreous samples of eyes with and without IVR were unpaired. Fibrovascular membranes from seven PDR patients were surgically retrieved during vitrectomy.

Vitreous samples were taken during the surgery and immediately centrifuged for 5 min at 4°C at 3,000 rotations per minute (rpm). The liquid component without sediment was immediately stored at −80°C until analysis. Fibrovascular membranes were immediately frozen at −80°C.

### ELISA Analysis

The concentrations of VEGF (Quantikine VEGF ELISA Kit; R&D Systems, Inc., Minneapolis, MN, USA) and EPO (Quantikine VEGF ELISA Kit; R&D Systems, Inc., Minneapolis, MN, USA) in the vitreous fluid were measured using enzyme-linked immunosorbent assay kits. Each assay was performed in accordance with the instructions of the user manual of the kit. Standard curves for each cytokine were generated using the reference cytokine concentrations supplied with the kit.

### Immunofluorescence Staining

Immunofluorescence staining was performed on the frozen sections of the FVMs by staining with the following antibodies: rabbit anti-EPO polyclonal IgG (1:150 dilution; No. ab126876 Abcam, Cambridge, MA, USA), mouse anti-CD105 monoclonal IgG (1:150 dilution; No. ab69772 Abcam, Cambridge, MA, USA), rabbit anti-VEGF polyclonal IgG (1:200 dilution; No. ab39250 Abcam, Cambridge, MA, USA), tetramethylrhodamine isothiocyanate- conjugated goat anti-mouse IgG (1: 200 dilution; Zhongshan Goldenbridge Biotechnology Co. Ltd., Beijing, China), and/or fluorescein isothiocyanate- conjugated goat anti-rabbit IgG (1: 200 dilution; Zhongshan Goldenbridge Biotechnology Co. Ltd.). The samples were counterstained with 4′,6′-diamino- 2-phenylindole (DAPI) (1: 1,000 dilution, No. D9542; Sigma-Aldrich, St. Louis, MO, USA). All the sections were examined using a fluorescence microscope (DS-Ril-U2; Nikon, Tokyo, Japan) and photographed (DS-U2; Nikon).

### Statistical Analysis

A statistical analysis was performed using GraphPad Prism 8 software. The data were presented as the mean ± standard deviation. T Differences between the study group and the control group were estimated with a non-parametric Mann-Whitney rank sum test and *t*-test when appropriate. Parameters were used Kruskal-Wallis *H*-test and ANOVA test to compare variables among various groups when appropriate. Chi-squared test or Fisher's exact test were used to compare non-continuous variables. Correlation coefficients were determined by using the Pearson correlation test on the transformed data of a decadic logarithm scale. Two-tailed probabilities of <0.05 were considered to indicate statistical significance.

## Result

### Demographic Data of Patients

The main characteristic of the 24 patients with PDR and 11 non-diabetic control patients enrolled in the study are shown in Table 1. The PDR group and control group showed no significant difference in gender. The mean age of 65.64 ± 5.14 in the control group was significantly older than that for the PDR without and with IVR group (54.71 ± 6.94, and 53.90 ± 7.95, respectively, *P* < 0.01).

**Table 1:** General clinical information of the patients.

**Variables**	**MH and ERM**	**PDR**		***P-value***	**P1**	**P2**	**P3**
	**(control group)**	**Non-IVR**	**IVR**		**#**	**§**	**&**
Patients, *n*	11	14	10	–			
Age (years)	65.64 ± 5.14	54.71 ± 6.94	53.90 ± 7.95	**0.0003**	0.001	0.001	0.95
Female/male	7/4	7/7	5/5	0.759	0.79	0.81	0.99
DM duration (y)	/	12.86 ± 4.43	16 ± 3.23	0.076			
Hypertension	4 (36.36%)	8 (57.14%)	4 (40%)	0.543	0.57	0.98	0.70
Visual acuity (LogMar)							
Before surgery	0.82 ± 0.57	1.97 ± 0.58	1.60 ± 0.86	**<0.001**	**<0.001**	**0.001**	0.577
After surgery	0.60 ± 0.29	1.27+0.39	1.49 ± 0.38	**<0.001**	**<0.001**	**<0.001**	0.327
Intraocular pressure							
Before surgery	13.27 ± 2.14	13.21 ± 3.49	14.20 ± 3.67	0.72	0.99	0.77	0.74
After surgery	12.27+1.90	17.00 ± 2.71	16.40 ± 4.19	**0.001**	**0.001**	**0.01**	0.88

*MH, macular hole; ERM, epiretinal membrane; DM, diabetes mellitus; PDR, proliferative diabetic retinopathy; ^#^Control vs. Non-IVR; ^§^Control vs. IVR; ^&^Non-IVR vs. IVR*.

*Bold value indicates p < 0.05, which are statistically significant*.

The duration of diabetes mellitus in Non-IVR group was 12.86 ± 4.43 years, and it was 16 ± 3.23 years in the IVR group. No statistically significant difference was noted in the duration of diabetes mellitus between the PDR groups (*P* = 0.076). As for hypertension history, the ration of the control group was 36.36%, which was lower than that for the PDR without (57.14%) and with IVR group (40%) (*P* = 0.54).

A statistically significant difference in the mean visual acuity (Log Mar) values were found among the three groups for both preoperative and postoperative vision. Before vitrectomy, the visual acuity values in the control group (0.82 ± 0.57) were significantly superior to the other two PDR groups (1.97 ± 0.58 and 1.60 ± 0.86, respectively, *P* < 0.01). After vitrectomy, visual acuity improved in all three groups. Similarly, visual acuity in the control group was better than that in the PDR group (*P* < 0.01).

The mean intraocular pressure (IOP) value before vitrectomy, measured using applanation tonometry in this study, was 13.27 mmHg in the MH+ERM group of subjects, 13.21 mmHg in the Non-IVR group and 14.20 mmHg in the IVR group. No statistically significant difference was found in the average IOP values among the three groups (*P* = 0.72). After vitrectomy, the IOP value in the PDR groups (17.00 ± 2.71 mmHg in without IVR group, 16.40 ± 4.19 mmHg in with IVR group) was higher than that in the control group (12.27 ± 1.90 mmHg) (*P* = 0.001).

### EPO and VEGF Levels in Vitreous

**Figure 1:**
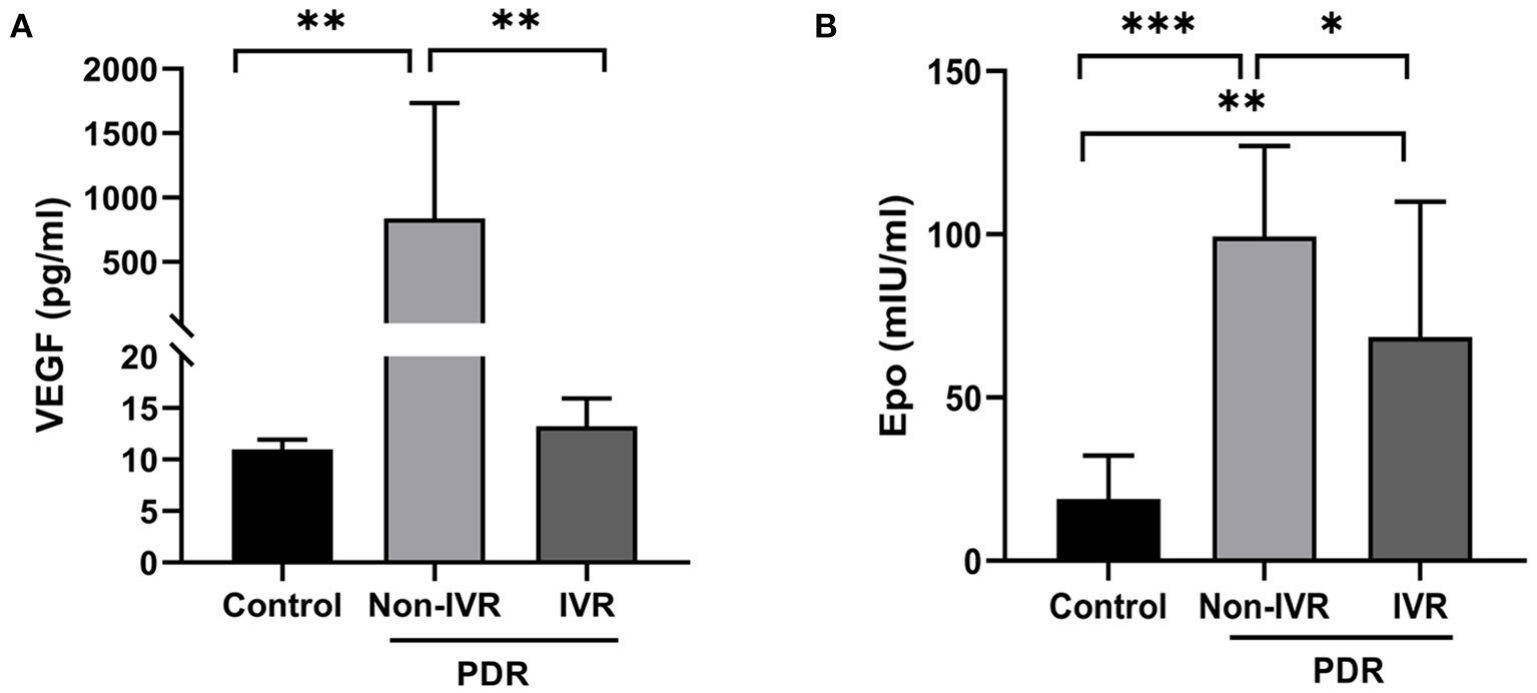
Vitreous concentration of VEGF **(A)** and EPO **(B)** in the control and PDR group. *P < 0.05; **P < 0.01; ***P < 0.0001. PDR, proliferative diabetic retinopathy; IVR, intravitreal ranibizumab.

Samples of undiluted vitreous fluid were collected from the eyes of 24 patients with PDR and from 11 patients in the control group. Table 2 presents the concentrations (medians and 95% CI) of EPO and VEGF in the vitreous fluid among the three groups. The median EPO level was 99.29 mIU per millilitre (95% CI: 83.25–115.3) in the patients with Non-IVR group and 68.57 mIU per millilitre (95% CI: 38.91–98.24) in the patients with IVR group and 18.96 mIU per millilitre (95% CI, 10.02–27.90) in the patients with Non-diabetic ocular diseases (*p* < 0.001). The median VEGF level was 836.3 pg/ml (95% CI: 316.90–1,356) in the patients with Non-IVR group and 13.22 pg/ml (95% CI: 11.27–15.17) in the patients with IVR group and 10.98 pg/ml (95% CI, 10.32–11.64) in the control group (*p* < 0.001). The vitreous EPO and VEGF levels were significantly higher in the patients with PDR than in the control group (Figure 1).

**Table 2:** Vitreous levels of erythropoietin and vascular endothelial growth factor (VEGF).

**Variables**	**MH and ERM**	**PDR**		***P-*** **value**	**P1**	**P2**	**P3**
	**(control group)**	**Non-IVR**	**IVR**		**#**	**§**	**&**
EPO (mIU/ml)							
Mean ± SD	18.96 ± 13.30	99.29 ± 27.77	68.57 ± 41.47	**<0.001**	**<0.001**	**0.001**	**0.04**
95% CI	10.02–27.90	83.25–115.3	38.91–98.24				
VEGF (pg/ml)							
Mean ± SD	10.98 ± 0.98	836.30 ± 899.5	13.22 ± 2.72	**0.001**	**0.003**	0.99	**0.004**
95% CI	10.32–11.64	316.90–1356	11.27–15.17				

*MH, macular hole; ERM, epiretinal membrane; DM, diabetes mellitus; PDR, proliferative diabetic retinopathy; ^#^Control vs. Non-IVR; ^§^Control vs. IVR; ^&^Non-IVR vs. IVR*.

*Bold value indicates p < 0.05, which are statistically significant*.

**Figure 2:**
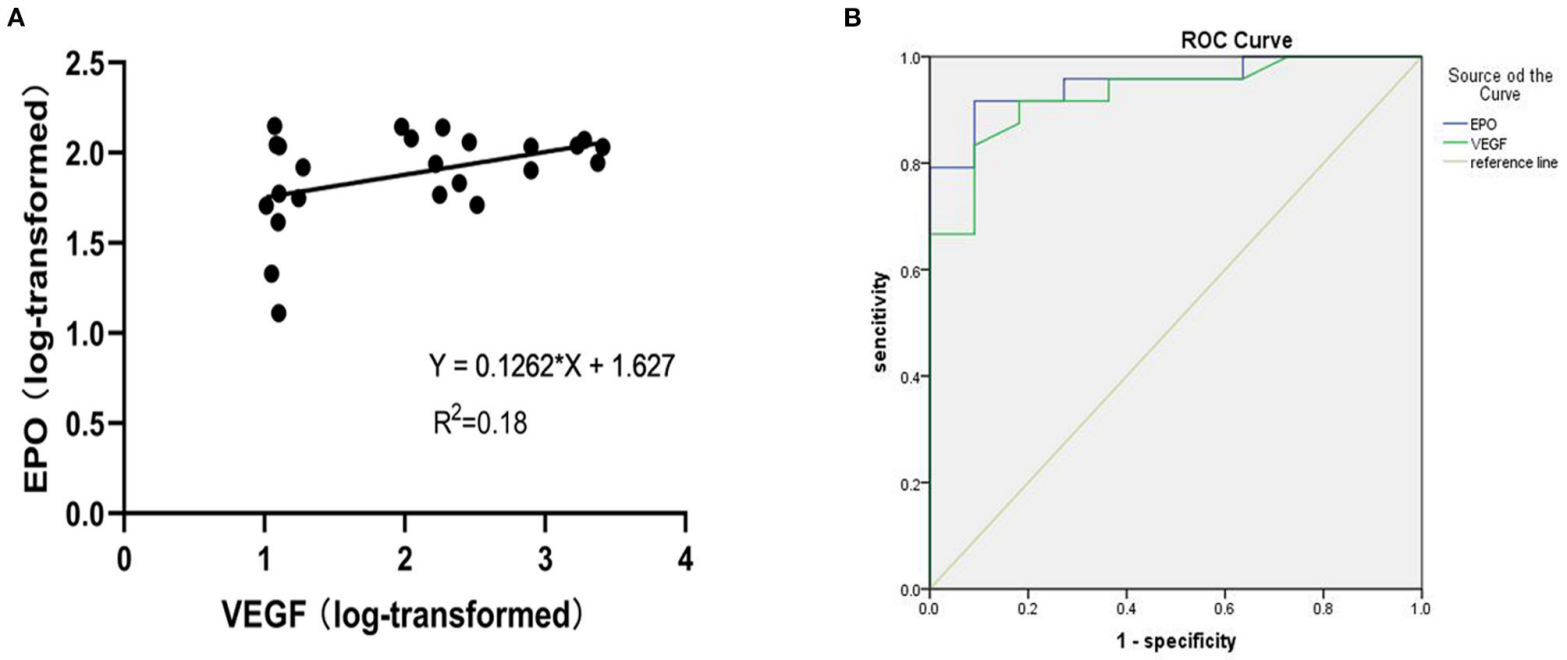
**(A)** Shows a scatter plot for the correlation between log-transformed vitreous VEGF and EPO levels in patients with PDR. **(B)** Receiver operating characteristic (ROC) curve of EPO and VEGF levels in PDR.

A scatter plot of the log-transformed levels of EPO and VEGF in the patients with PDR indicated a positive correlation (Figure 2A). However, the Pearson correlation coefficient was 0.18, and no significant difference was seen (*P* = 0.22).

For exploratory purposes, we also analysed the receiver operating characteristic (ROC) curve of the EPO and VEGF levels in the PDR group (Figure 2B). The AUC values for EPO and VEGF were 0.951 and 0.938, respectively.

### Immunofluorescence Staining

**Figure 3:**
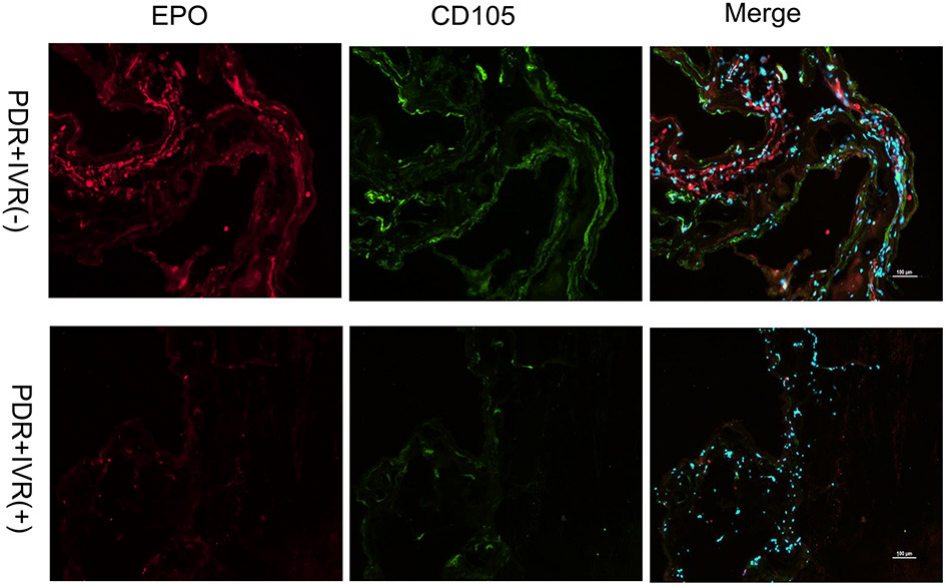
Epiretinal fibrovascular membranes (FVMs) showed strong positive for EPO and the marker CD105 of neovascular endothelial cells in the Non-IVR group. Scale bars: 100 μm.

**Figure 4:**
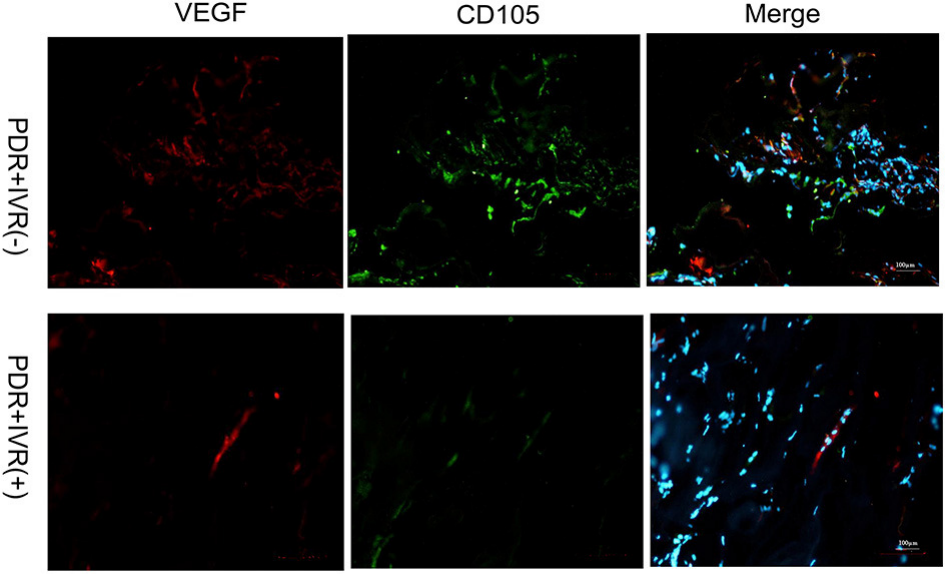
Epiretinal fibrovascular membranes (FVMs) showed strong positive for VEGF and the marker CD105 of neovascular endothelial cells in the Non-IVR group. Scale bars: 100 μm.

**Figure 5:**
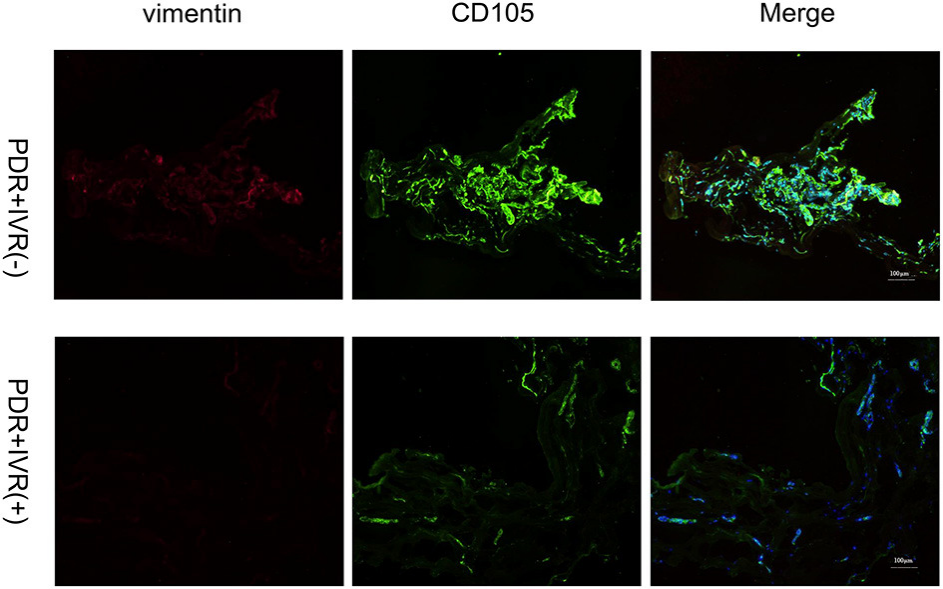
Epiretinal fibrovascular membranes (FVMs) showed negative for Vimentin and positive for the marker CD105 of neovascular endothelial cells in the Non-IVR group. Scale bars: 100 μm.

The staining of epiretinal fibrovascular membranes (FVMs) showed strong positives for EPO and VEGF and for the marker CD105 of the neovascular endothelial cells in the Non-IVR group (Figure 3 top row and Figure 4 top row). After IVR, the positive expressions significantly decreased for EPO, VEGF, and CD105 in the FVMs of PDR patients (Figure 3 bottom row and Figure 4 bottom row). Figure 5 reveals the expression of vimentin for the maker of fibroblastic cells, which was weakly positive in the PDR group and negative in the IVR group (Figure 5).

## Discussion

The present study indicates that the vitreous levels of EPO and VEGF in PDR patients is strikingly higher than the levels in Non-diabetic patients. These results are consistent with other researchers (11–17). Furthermore, the levels of VEGF and EPO in vitreous fluids and FVMs significantly declined after IVR. Although not significant, the vitreous levels of EPO showed a trend of positive correlation with VEGF in the DR patients. The ROC curve analysis showed that EPO and VEGF had clear specificity and sensitivity in the indication of PDR. Our present study provides a valuable foundation for further study of the relationship between EPO and VEGF in PDR and their potential future use in clinical practise.

VEGF-mediated pathogenic effects are primarily related to vascular permeability and neovascularization (18). VEGF was found to be closely related to the development and progression of PDR (11, 19, 20). Recently, anti-VEGF therapy has led to great advances in ocular neovascular diseases. However, the inhibition of VEGF is not associated with a total regression of retinal neovascularization, indicating that other angiogenic factors and inflammation factors may play a role in this process, including TNF-α, IL-6, EPO and the pigment epithelium-derived factor (PEDF) (11).

Many recent studies have shown that EPO has different biological effects *in vivo* and *in vitro* studies (13, 21). EPO was demonstrated to protect against the VEGF-induced permeability of the blood-brain barrier (BBB) through restoring the tight junction proteins and VE-cadherin in experimental diabetic retinopathy and *in vitro* bovine model (22, 23). EPO improved oxygen carriage to retinal tissue and ameliorated diabetic retinopathy. However, some studies have indicated that EPO has an angiogenic potential equivalent to that of VEGF and independently contributes to retinal neovascularization in the pathogenesis of PDR. Therefore, the precise role of EPO is still of great interest for many researchers. It is yet unclear whether EPO has a protective or aggravating role in DR, considering the contradictive results from various studies.

Our study demonstrated a high level of EPO in vitreous fluids and FVMs in PDR patients, which was consistent with other studies about EPO. However, the source of the locally increasing EPO in PDR was not confirmed in the present study since the serum samples from the PDR patients were not collected due to ethical consideration. Thus, we were unable to explore the possible association between serum EPO and vitreous EPO in PDR patients. In a previous study, Semeraro et al. and Watanabe et al. showed that serum EPO concentrations did not significantly differ between diabetic and non-diabetic patients (11, 13). Furthermore, there was no correlation between haemoglobin and intraocular EPO deposition (in both vitreous and aqueous humours), but positive correlation between EPO and glycated haemoglobin and hyperglycaemia was confirmed. It seems clear that EPO in vitreous fluid is probably not due to the breakdown of the blood retinal barrier and is not serum derived. Hernandez first detected EPO RNA expression in the adult human retina, and its expression was significantly higher in diabetic than in non-diabetic donors. EPO expression was found to be more abundant in RPE than in the neuroretina, which supported the notions that EPO is actually produced in the local microenvironment of the eye and that ischemia and hypoxia caused by hyperglycaemia may be stimulating factors (16).

We further explored the relationship between EPO and VEGF, especially EPO after anti-VEGF treatment. Our result revealed that the levels of EPO and VEGF increased in vitreous fluid in the PDR patients. In addition, the level of EPO showed a trend of positive correlation with the VEGF level, yet no significant correlation was found. This is consistent with the results of other studies, Semeraro et al. found no correlation between the concentrations of EPO and VEGF in the vitreous body (11). Recently, anti-VEGF therapy has become the first-line treatment in PDR-complicated neovascularization and DME. However, EPO changes after anti-VEGF treatment have not been evaluated. Our study, for the first time, demonstrated that the concentration of EPO in vitreous fluid and FMVs significantly decreased after anti-VEGF treatment, indicating a possible interaction between EPO and VEGF in PDR. EPO and VEGF may be involved in similar signalling pathways. However, further studies are needed to verify these hypotheses.

Due to the contradictive results of EPO from various studies, we explored whether EPO has a protective or aggravating role in PDR. It was suggested that serum EPO concentrations increased in direct proportion with the severity of the clinical stage of PDR and that blocking EPO may be beneficial to the treatment of PDR (13). However, in an early diabetes animal model and DME patients, exogenous EPO administration not only protected against the VEGF-induced permeability of the BBB and restored the tight junction proteins, but it also counteracted neurodegeneration (16, 20, 24). Another *in vivo* study confirmed that compared to IVB alone with intravitreal IVB, IVB combined with EPO did not significantly improve visual acuity and reduce retinal thickness in DME patients, nor did any retinopathy progression or neovascularization (25). Therefore, EPO may play different roles in different stages of DR. It is important to find a dynamic balance for EPO between the protection effect on the permeability of the BBB and the risk for retinal vaso-proliferative diseases. Further studies are necessary, including research on the effect of angiogenesis on exogenous EPO and neuronal side effects and the BBB permeability of an EPO blockade.

Furthermore, we analysed the receiver operating characteristic (ROC) curves of EPO and VEGF. The ROC curve is a useful tool for evaluating the performance of diagnostic tests within the range of possible values of predictive variables. The area under the ROC curve (AUC) is an overall measure of a test's ability to determine whether a particular situation exists or not. An AUC of 0.5 indicates a test with no discrimination (i.e., no better than chance), while an AUC of 1.0 indicates a test with perfect discrimination (26, 27). The AUCs of EPO and VEGF were 0.951 and 0.928, respectively, which suggested that EPO and VEGF have equal diagnostic value in the prediction of PDR. Of course, the detection of vitreous body fluid is an invasive examination, which is difficult to pass in an ethics review. But the results of ROC curve in this study indicated that both EPO monitoring and VEGF are of great significance, which may be of certain significance for the treatment of different stages of clinical DR.

Our study has some limitations. First, this was a retrospective comparative study with a limited number of patients enrolled. Second, paired samples of vitreous fluid from the same eye (before and after the injection) were not collected due to ethical considerations. Therefore, only the vitreous fluid of post-injection eyes was examined, based on the comparability of the groups. Third, the grouping of DR was not further refined. In particular, NPDR patients with or without DME were not included in this study. Because vitrectomy is not needed in patients with NPDR and DME, no vitreous and intraocular fluid samples could be collected due to ethical considerations. Finally, further research on the mechanisms of the different effects of EPO in DR is needed.

In summary, we found that both of the VEGF and EPO level were significantly increased in PDR patients, which have equal diagnostic value in the prediction of PDR. IVR could reduce the EPO level, but not enough to the normal level. The interaction between EPO and VEGF still needs to be further explored.

## Data Availability Statement

The original contributions presented in the study are included in the article/supplementary material, further inquiries can be directed to the corresponding authors.

## Ethics Statement

The studies involving human participants were reviewed and approved by Ethical Review Committee of Beijing Chaoyang Hospital. The patients/participants provided their written informed consent to participate in this study.

## Author Contributions

Design and conduct of the study by LC, JF, and YT. Collection, management, analysis, and interpretation of the data by LC, JF, YS, FL, FM, YW, WY, and YT. Preparation of the manuscript by LC and JF. Review and final approval of the manuscript by all the authors.

## Funding

This study was supported by National Natural Science Foundation of China (Nos. 82070948 and 82101142), Beijing Talent Project (No. 2020027), Shunyi District Beijing Science and technology achievements transformation coordination and service platform construction fund (SYGX202010), and Beijing Hospitals Authority Youth Programme (No. QML20190303).

## Conflict of Interest

The authors declare that the research was conducted in the absence of any commercial or financial relationships that could be construed as a potential conflict of interest.

## Publisher's Note

All claims expressed in this article are solely those of the authors and do not necessarily represent those of their affiliated organizations, or those of the publisher, the editors and the reviewers. Any product that may be evaluated in this article, or claim that may be made by its manufacturer, is not guaranteed or endorsed by the publisher.
